# A review of Cochrane reviews on pharmacological treatment for attention deficit hyperactivity disorder

**DOI:** 10.1590/1980-57642021dn15-040001

**Published:** 2021

**Authors:** Giovanna Marcílio Santos, Elaine Marcílio Santos, Gustavo Duarte Mendes, Yara Dadalti Fragoso, Mariani Rafaela Souza, Ana Luiza Cabrera Martimbianco

**Affiliations:** 1 Universidade Metropolitana de Santos – Santos, SP, Brazil.; 2 Postgraduate Program in Health and Environment, Universidade Metropolitana de Santos – Santos, SP, Brazil.

**Keywords:** attention deficit hyperactivity disorder, systematic review, desipramine, amphetamine, methylphenidate, transtorno do déficit de atenção com hiperatividade, revisão sistemática, desipramina, anfetamina, metilfenidato

## Abstract

Attention deficit hyperactivity disorder (ADHD) is one of the most frequent childhood psychiatric problems.

**Objective:**

The objective of this study was to identify, synthesize the results, and critically evaluate all Cochrane systematic reviews (SRs) on the pharmacological interventions for children and adolescents (up to age 18) diagnosed with ADHD.

**Methods:**

The search was performed in the *Cochrane Database of Systematic Reviews* (via Wiley) in July 2020.

**Results:**

The search strategy resulted in four SRs of high methodological quality, analyzing 51 randomized clinical trials (9,013 participants). Compared to placebo, treatment with tricyclic antidepressants (TCAs) (desipramine), amphetamine, and methylphenidate showed improvement in symptoms such as difficulty concentrating, impulsivity, and hyperactivity in the short term (up to 6 months). There was an increase in the occurrence of adverse events, such as reduced appetite, difficulty sleeping, and abdominal pain. Insufficient evidence was found to support the effects of supplementation with polyunsaturated fatty acids.

**Conclusions:**

The use of TCAs, amphetamine, and methylphenidate in children and adolescents with ADHD seems to present positive effects and higher rates of minor adverse events when compared to placebo.

## RESUMO.

Déficit de atenção e hiperatividade (TDAH) é uma das mais frequentes condições psiquiátricas da infância.

**Objetivo:**

O objetivo deste artigo foi identificar, sintetizar os resultados e avaliar criticamente todas as revisões sistemáticas (RS) da Cochrane sobre as intervenções farmacológicas para crianças e adolescentes (até 18 anos de idade) diagnosticados com transtorno do déficit de atenção com hiperatividade.

**Métodos:**

A pesquisa foi realizada na base de dados Cochrane de Revisões Sistemáticas — CDSR (via Wiley) em julho de 2020.

**Resultados:**

A estratégia de busca resultou em quatro RS de alta qualidade metodológica, que analisavam 51 ensaios clínicos randomizados (9.013 participantes). Comparado ao placebo, o tratamento com antidepressivos tricíclicos (desipramina), anfetamina e metilfenidato apresentou melhora nos sintomas, como dificuldade de concentração, impulsividade e hiperatividade no curto prazo (até seis meses). Houve aumento na ocorr≖ncia de eventos adversos, como redução do apetite, dificuldade para dormir e dor abdominal. Foram encontradas evid≖ncias insuficientes para apoiar os efeitos da suplementação com ácidos graxos poli-insaturados.

**Conclusões:**

Com base nos resultados de revisões sistemáticas Cochrane, o uso de antidepressivos tricíclicos, anfetamina e metilfenidato em crianças e adolescentes com TDAH parece apresentar efeitos positivos e taxas mais elevadas de eventos adversos menores quando comparado ao placebo. Dado o alto risco de viés nos estudos primários incluídos nessas RS, ainda são necessários novos ensaios clínicos randomizados com rigor metodológico para apoiar esses achados.

## INTRODUCTION

Attention deficit hyperactivity disorder (ADHD) is one of the most diagnosed and treated childhood psychiatric problems. The worldwide prevalence of ADHD in children and adolescents varies between 5.3 and 9.4%,^1,2,3^ with a predominance in males and ages 2–5 years.^4,5^ Depending on the classification system used for diagnosis, these aspects may vary slightly.^3,6^

Individuals with ADHD have a neurodevelopmental disorder, showing cognitive deficits and a significant deficiency in the family, social, and academic spheres. These children and adolescents have levels of inattention, impulsivity, or hyperactivity, either in a separate or associated manner, which are not appropriate for their age.^1,3,7^ Their difficulty in planning, solving problems, and maintaining attention and memory adds to factors such as motivational delays and mood dysregulation. Altogether, these aspects result in lasting effects on the lives of these individuals, leading to worse quality of life.^7^

ADHD symptoms improve significantly with an appropriate treatment. For it to be effective, a well-elaborated and specific plan is needed to describe treatment methods and goals, as well as means of monitoring care over time and specific follow-up plans. In most cases, associated pharmacological and nonpharmacological interventions (e.g., behavioral and psychosocial therapies) are recommended.^1,8^ The National Institute of Clinical Excellence recommends that clinicians consider the presence of coexisting conditions, potential side effects, cognitive ability, drug abuse, cost–benefit, and the preferences of parents and/or patients.^8^ Drugs considered first line to treat ADHD symptoms include methylphenidate and amphetamine psychostimulants, followed by nonstimulants (atomoxetine and alpha-agonists: clonidine and guanfacine).^9^ Stimulant drugs seem to have a potential protective effect on functional and behavioral outcomes, especially in adolescents with ADHD.^10^ In addition, as the deficiency of polyunsaturated fatty acids (omega-3 and omega-6) seems to be associated with a potential pathogenic mechanism in ADHD, some studies recommend this supplement as an adjuvant.^11^

Given the wide range of pharmacological interventions available for the treatment of ADHD and the high number of studies published in this regard, it is necessary to systematically synthetize all high-quality evidence. Summarizing the results of these studies in a single document helps the decision-making by the health professional. Thus, the purpose of this review was to identify, synthesize the results, and critically evaluate all Cochrane systematic reviews (SRs) on the pharmacological interventions for children and adolescents diagnosed with ADHD.

## METHODS

This review of SRs followed the recommendations of the *Cochrane Handbook for Systematic Review of* Interventions.^12^

### Criteria for inclusion of systematic reviews

We included all SRs published by Cochrane on pharmacological treatment for children (up to 12 years) and adolescents (up to 18 years, not adults) diagnosed with ADHD. There was no restriction on the date of publication of the review. SR protocols and those marked as “withdrawn” from the Cochrane Library were excluded. SRs addressing any pharmacological intervention, regardless of dose, duration, and frequency of treatment, compared to placebo, no treatment, or other interventions, were included. Clinical outcomes, such as symptom improvement and safety (report of adverse events), were analyzed.

### Search strategy for the identification of systematic reviews

The search was performed in the *Cochrane Database of Systematic Reviews* (via Wiley), on July 28, 2020. The search strategy used the Mesh term “Attention Deficit Disorder with Hyperactivity” and synonyms.

### Selection of studies and data extraction

The identified SRs were independently selected by two researchers (GMS and ALCM), who analyzed the eligibility of reviews by reading titles and abstracts. The eligible studies were then evaluated in full text and classified as included or excluded studies. Divergences were decided by consensus. The selection process was done through the Rayyan online platform.^13^

The included SRs had their data extracted by two independent researchers (GMS and EMS), through a standardized form with information on methodological characteristics of the reviews, characteristics of the participants, and results of the outcomes evaluated.

#### Methodological quality assessment

SRs were evaluated for their methodological quality using the AMSTAR-2 tool (Assessing the Methodological Quality of Systematic Reviews),^14^ which was applied by two researchers (GMS and EMS) independently. The AMSTAR-2 is a tool composed of 16 items related to the research question, study planning, justification for the choice of the design of the included primary study, search strategies, selection of studies and extraction of data in pairs, description of excluded studies, characteristics of the included studies, methods of risk of bias assessment, sources of funding, methods used to combine the results in meta-analysis, assessment of the impact of the risk of bias in the meta-analyses and discussion, explanation of heterogeneity, investigation of publication bias, and declaration of conflict of interest of authors. Each item is classified as appropriate (“yes”), partially adequate (“partially yes”), inadequate (“no”), or not applicable (“NA”). AMSTAR-2 uses a structure based on the 16 items to classify the SR according to the following degrees of confidence: critically low, low, moderate, and high. The evaluation used the checklist available on the AMSTAR-2 website (http://amstar.ca/Amstar_Checklist.php).

#### Data analysis

The results of the included SRs were presented narratively, considering the quality of the studies evaluated by AMSTAR-2. As the included reviews addressed different interventions, it was not necessary to analyze the overlapping of results, that is, when the same primary study was included in more than one SR.

## RESULTS

The search strategy resulted in 22 Cochrane SRs. However, after reading the titles and abstracts, 16 were excluded since they did not meet the inclusion criteria. Thus, four SRs were included and analyzed in this review.^15 16,17,18^

The four SRs were published between 2012 and 2016 and assessed different medications for the treatment of ADHD in children and adolescents. The main features of the included SRs included are detailed in the subsequent section.

### Methylphenidate

This SR^15^ evaluated the benefits and harms of methylphenidate for children and adolescents with ADHD. It included 38 randomized clinical trials (RCTs) with parallel design (5,111 participants) and 147 with crossover design (7,134 participants). Age ranged from 13 to 18 years, there was a predominance of males (5:1), and the duration of treatment was 75 days. Table 1 presents the main results of the meta-analyses performed in this SR. The results of the meta-analyses suggest that methylphenidate may improve the ADHD symptoms reported by the teacher, the general behavior reported by the teacher, and the quality of life reported by parents. However, the low certainty evidence evaluated by the GRADE approach due to high risk of bias, imprecision, and inconsistency means that we cannot be sure of the magnitude of the effects. A previous version of the SR^19^ had been incorporated into the newer one.^15^

**Table 1.** Summary of the main outcomes: methylphenidate for attention deficit hyperactivity disorder.

Methylphenidate *versus* placebo	
Symptom improvement (evaluated by teachers)	Improvement in favor of methylphenidate (SMD −0.77; 95%CI −0.90 to −0.64; 19 RCTs; 1,698 participants)
Overall behavior improvement (evaluated by teachers)	Improvement in favor of methylphenidate (SMD −0.87, 95%CI −1.04 to −0.71; 5 RCTs; 668 participants)
Improvement in quality of life (assessed by parents)	Improvement in favor of methylphenidate(SMD 0.61, 95%CI 0.42–0.80; 3 RCTs; 514 participants)
Serious adverse events (mortality, hospitalization)	No difference between groups, but there was a wide CI (RR 0.98, 95%CI 0.44–2.22; 9 RCTs; 1,532 participants)
Mild adverse events	29% increased risk of adverse events in the methylphenidate group (RR 1.29, 95%CI 1.10–1.51; 21 RCTs; 3,132 participants)
Difficulty sleeping	60% increased risk in methylphenidate group (RR 1.60, 95%CI 1.15–2.23; 13 RCTs; 2,416 participants)
Reduced appetite	266% increased risk methylphenidate group (RR 3.66, 95%CI 2.56–5.23; 16 RCTs; 2,962 participants)

SMD: standardized mean difference; 95%CI: 95% confidence interval; RCT: randomized clinical trial; RR: relative risk.

### Amphetamine

The objective of this SR^16^ was to evaluate the efficacy and safety of amphetamines for ADHD in children and adolescents. Twenty-three RCTs totaling 2,675 participants between the ages of 3 and 17 years and mean duration of treatment of 28 days were included. The certainty of the body of evidence was classified by the GRADE approach as very low to low due to a high risk of bias for most of the included studies and the presence of substantial heterogeneity, indicating that the results should be interpreted with caution and that future methodologically rigorous studies are still needed. Table 2 presents the results of the main meta-analyses related to the evaluation of symptom improvement and the occurrence of adverse events. Although amphetamine seems effective in reducing ADHD symptoms in the short term, some related adverse events have been observed. Future studies should monitor patients in the long term (more than 12 months), include psychosocial outcomes (e.g., quality of life), and have greater methodological rigor.

**Table 2.** Summary of the main outcomes: amphetamine for attention deficit hyperactivity disorder.

Amphetamine *versus* placebo	
Symptom improvement (assessed by parents)	Improvement in favor of amphetamine (SMD −0.57; 95%CI −0.86 to −0.27; 7 RCTs; 1,247 participants)
Symptom improvement (assessed by teachers)	Improvement in favor of amphetamine(SMD −0.55; 95%CI −0.83 to −0.27; 5 RCTs; 745 participants)
Symptom improvement (assessed by clinicians)	Improvement in favor of amphetamine (SMD −0.84; 95%CI −1.32 to −0.36; 3 RCTs; 813 participants)
Mild adverse events (at least one adverse event)	30% increased risk of adverse events in the amphetamine group (RR 1.30; 95%CI 1.18–1.44; 6 RCTs; 1,742 participants)
Difficulty sleeping	280% increased risk of adverse events in the amphetamine group (imprecise effect – wide CI) (RR 3.80; 95%CI 2.12 to 6.83; 10 RCTs; 2,429 participants)
Reduced appetite	531% increased risk of adverse events in the amphetamine group (imprecise effect – wide CI)(RR 6.31; 95%CI 2.58–15.46; 11 RCTs; 2,467 participants)
Abdominal pain	44% increased risk of adverse events in the amphetamine group (imprecise effect – wide CI) (RR 1.44; 95%CI 1.03–2.00; 10 RCTs; 2,155 participants)

SMD: standardized mean difference; 95%CI: 95% confidence interval; RCT: randomized clinical trial; RR: relative risk.

### Tricyclic antidepressants

The objective of this SR^17^ was to evaluate the efficacy of tricyclic antidepressants (TCAs) in reducing hyperactivity, impulsivity, and inattention in children and adolescents diagnosed with ADHD. Six RCTs with a total of 216 participants, between 6 and 18 years old, were included. The duration of treatment ranged from 2 to 9 weeks. These RCTs evaluated the effects of three TCAs (desipramine, clomipramine, and nortriptyline) on the main symptoms of ADHD. The certainty of evidence evaluated by the GRADE approach varied from very low to low, due to methodological limitations of RCTs, small sample size, wide confidence interval, and heterogeneity between included studies. The results of the main meta-analyses are described in Table 3. Symptom improvement outcomes were evaluated using ADHD-related behavioral change scales and treatment response rates. Overall, the results from meta-analyses showed beneficial effects in favor of TCAs, but for some outcomes, these estimated effects were imprecise due to a wide confidence interval.

**Table 3.** Summary of the main outcomes: tricyclics for attention deficit hyperactivity disorder.

Desipramine *versus* placebo	
Symptom improvement (assessed by parents)	Improvement in favor of desipramine(SMD −1.42; 95%CI −1.99 to −0.85; 2 RCTs; 99 participants)
Symptom improvement (assessed by teachers)	Improvement in favor of desipramine (SMD −0.97; 95%CI −1.66 to −0.28; 2 RCTs; 89 participants)
Symptom improvement (assessed by clinicians)	Improvement seems to be in favor of desipramine, but these results are imprecise (wide CI) (OR 26.41; 95%CI 7.41–94.18; 2 RCTs; 103 participants)
Treatment interruption	No difference between groups, but there was a wide CI and substantial heterogeneity (I^2^=66%)(RD −0.10; 95%CI −0.25 to 0.04; 3 RCTs; 134 participants)

Nortriptyline *versus* placebo	
Symptom improvement (assessed by clinicians)	Improvement seems to be in favor of desipramine, but these results are imprecise (wide CI) (RR 7.88; 95%CI 1.10–56.12; 1 RCT; 22 participants)

Desipramine *versus* clonidine (in patients with ADHD plus Tourette’s syndrome)	
Symptom improvement (assessed by parents)	Improvement in favor of desipramine (SMD −0.90, 95%CI −1.40 to −0.40; 1 RCT; 68 participants)

SMD: standardized mean difference; 95%CI: 95% confidence interval; RCT: randomized clinical trial; OR: *Odds Ratio*; RR: relative risk; RD: risk difference; ADHD: attention deficit hyperactivity disorder.

Although no serious adverse events have been identified in patients using desipramine, there have been slight increases in diastolic blood pressure and heart rate. In addition, patients treated with desipramine had higher appetite reduction rates compared to placebo, while nortriptyline resulted in weight gain. The results of this SR suggest that, in the short term, desipramine improves ADHD symptoms, but its cardiovascular effects continue to be a clinical concern.

### Polyunsaturated fatty acids (omega-3 and omega-6)

The aim of this SR^18^ was to analyze the efficacy of the use of omega-3 and omega-6 in the treatment of ADHD in children and adolescents, compared to other treatments or placebo. Three RCTs with a total of 1,011 participants, aged between 6 and 18 years, were included. The duration of treatments ranged from 4 to 16 weeks. The risk of bias was classified as unclear for most RCTs because the methods of randomization and allocation concealment were not adequately described. There was a high risk of attrition bias because eight RCTs had lost participants during follow-up and did not perform intention-to-treat analysis. The results of meta-analyses for the improvement of symptoms (lack of attention, hyperactivity/impulsivity, socialization), quality of life, adverse events (dermatitis, diarrhea, gastrointestinal discomfort, among others), and losses during follow-up are detailed in Table 4. There was little evidence that fatty acid supplementation provided any benefit to the improvement of ADHD symptoms in children and adolescents. However, no adverse effects resulting from the intervention were identified. It is important that future RCTs present adequate sample sizes, use reliable selection criteria, and avoid the potential methodological biases identified in the clinical trials included in this SR.

**Table 4.** Summary of the main outcomes: polyunsaturated fatty acids for attention deficit hyperactivity disorder.

Omega-3 plus omega-6 *versus* placebo	
Symptom improvement (reported by the patient)	Improvement seems to be in favor of omega-3/6, but these results are imprecise (wide CI)(RR 2.19; 95%CI 1.04 to 4.62; 2 RCTs; 97 participants)
Symptom improvement (reported by parents)	No difference between groups (SMD −0.17; 95%CI −0.38 to 0.03; 5 RCTs; 413 participants)
Symptom improvement (reported by teachers)	No difference between groups (SMD 0.05; 95%CI −0.18 to 0.27; 4 RCTs; 324 participants)
Quality of life	No difference between groups (MD −0.12; 95%CI -3.71 to 3.47; 1 RCT, 138 participants)
Follow-up losses	No difference between groups (RR 0.95; 95%CI 0.69 to 1.31; 7 RCTs, 589 participants)

CI: confidence interval; RR: relative risk; 95%CI: 95% confidence interval; SMD: standardized mean difference; RCT: randomized clinical trial; MD: mean difference.

### Methodological quality assessment of the included systematic reviews

The methodological quality of the included SRs was evaluated by the AMSTAR-2 tool, and because these are Cochrane SRs whose methodology is rigorous and transparent, all were classified as high quality. Supplementary file presents the details of the assessment.

## DISCUSSION

This review included four SRs published by Cochrane on different pharmacological interventions for the treatment of children and adolescents with ADHD. Compared to placebo, the three drugs analyzed (TCA, amphetamine, and methylphenidate) showed improvement in symptoms reported by parents, clinicians, and teachers, especially in relation to difficulty concentrating, impulsivity, and hyperactivity. However, there was an increase in the occurrence of adverse events common to these medications, such as reduced appetite, difficulty sleeping, and abdominal pain. Considering the use of polyunsaturated fatty acid supplementation (omega-3 and omega-6), there is little evidence of benefits or risks. It is important to note that some outcomes analyzed presented an imprecise estimated effect due to a wide confidence interval. It probably occurred due to the small sample size and heterogeneity between RCTs.

Serious adverse events occurred to a lesser extent, but it is suspected that these results may have been underreported in the studies included in the reviews, for example, in the methylphenidate review, in which only 9 of the 185 clinical trials included reported this outcome. According to the study by Hennissen et al.,^20^ undesirable effects such as increased blood pressure and heart rate caused by medications such as amphetamine or methylphenidate in children can be interpreted as risk factors for cardiovascular diseases in adulthood; for this reason, these patients should be monitored periodically. Storebø et al.^19^ reported results from an SR of non-RCTs and suggested that methylphenidate may be associated with a series of serious adverse events in children and adolescents, which lead to discontinuation of treatment. However, the certainty of the evidence was considered very low, given the heterogeneity and poor methodological quality of the studies included (unclear to high risk of selection, attrition, reporting performance, and detection bias), and it was not possible to accurately estimate the true risk of adverse events. The authors report the need to identify the most susceptible individuals, in addition to conducting new RCTs of higher quality and with long-term analysis.

In the present review, most clinical trials included in the reviews analyzed short-term outcomes with a maximum follow-up time of 3 months, a fact that may have been influenced by the cross-design of many of the included studies. Thus, it was not possible to verify the effects of a longer exposure on the overall behavior of children and whether any beneficial effects can be decreased or compensated by an increased risk of harm. Short-term clinical trials are particularly problematic for chronic diseases such as ADHD, as children are likely to use stimulant medications for longer periods than those studied. In view of the above, the clinical decision to start and continue with treatment should consider the balance between the improvement of symptoms and reports of adverse events, in an individualized and careful way, since the lack of sleep, for example, can impact the quality of life and the child’s ability and learning.

Although the SRs of this review include many clinical trials, the methodological quality of these studies was classified as low to very low, a fact that compromises confidence in the estimates of effect and robustness of the evidence. Criteria such as the absence of allocation concealment and blinding of participants and outcome assessors can directly impact the results, especially on subjective outcomes that depend on the participants’ reports, and may be consciously or unconsciously modified in view of the knowledge of the allocation to the placebo group. Another relevant point to be considered is the uncertainty regarding the assessment reported by parents, which may present a certain degree of incoherence depending on social exposure.

A recent network meta-analysis^1^ provided updated evidence on drug treatment for ADHD, supporting the use of methylphenidate for children and adolescents and amphetamine for adults as the first choice of short-term treatment. The authors also described the need for further clinical trials to evaluate long-term effects of these drugs.

It is worth mentioning that despite approval of some drugs (and not others) to treat ADHD by North American and European agencies, not all countries have access to all drugs. Therefore, the unexpensive tricyclic molecules may be used in developing countries to treat ADHD. The present review focused on describing the efficacy and safety of any drug indicated for ADHD according to high-quality evidence.

In view of this scenario, the four SRs included in this review indicated the need for new RCTs, with adequate methodology based on CONSORT statement (Consolidated Standards of Reporting Trials),^21^ and higher sample sizes. It is recommended that the comparison be made between the drug of interest and placebo or another first-line drug, with evaluation of outcomes such as clinical improvement of symptoms by clinicians and/or teachers, using validated scales, and the occurrence of adverse events in the long term to analyze both the effectiveness and safety of interventions.

Based on the results of Cochrane SRs with high methodological quality, the use of TCAs, amphetamine, and methylphenidate showed improvement in symptoms of children and adolescents with ADHD compared to placebo. There was an increase in the occurrence of adverse events, such as reduced appetite, difficulty sleeping, and abdominal pain. Considering the use of polyunsaturated fatty acid supplementation (omega-3 and omega-6), there is little evidence of benefits or risks. Given the high risk of bias in the primary studies included in these reviews, new RCTs with methodological rigor are still needed to support these findings. Future clinical trials should evaluate long-term outcomes, in addition to measuring the impact of treatment on the quality of life of children and adolescents with ADHD.
